# Tremor Drugs in the Crosshairs

**DOI:** 10.5334/tohm.664

**Published:** 2021-11-26

**Authors:** Tjerk J. Lagrand, Alexander C. Lehn

**Affiliations:** 1 Department of Neurology, Princess Alexandra Hospital, Woolloongabba, Brisbane, Queensland, Australia; 2 University of Queensland, Brisbane, Queensland, Australia

**Keywords:** Tremor, alopecia areata, medication-induced, levodopa, propranolol, topiramate

## Abstract

**Background:**

Alopecia areata is a rare but debilitating adverse effect of drugs used in the treatment of tremors. Recurrent hair loss after different types of tremor medications has never been described before.

**Case Report:**

We herein report the case of a 56-year-old tremor patient who we diagnosed with tremor-dominant Parkinson’s disease. Unfortunately, she developed acute alopecia areata following the introduction of firstly levodopa/benserazide, secondly propranolol, and thirdly topiramate.

**Discussion:**

Our case report highlights alopecia areata as a possible side effect to a variety of drugs commonly used for tremor management. Fortunately, in most reported cases, as well as in our case, the hair loss is reversible.

## Introduction

Alopecia areata refers to the development of typical patches of hair loss, mainly from the scalp. This type of hair loss can be caused by a variety of medications, including several commonly used drugs for the treatment of tremors. Here we describe a unique case of a lady who unfortunately developed recurrent drug-induced alopecia after three different types of tremor medications.

## Case Description

**Figure 1:**
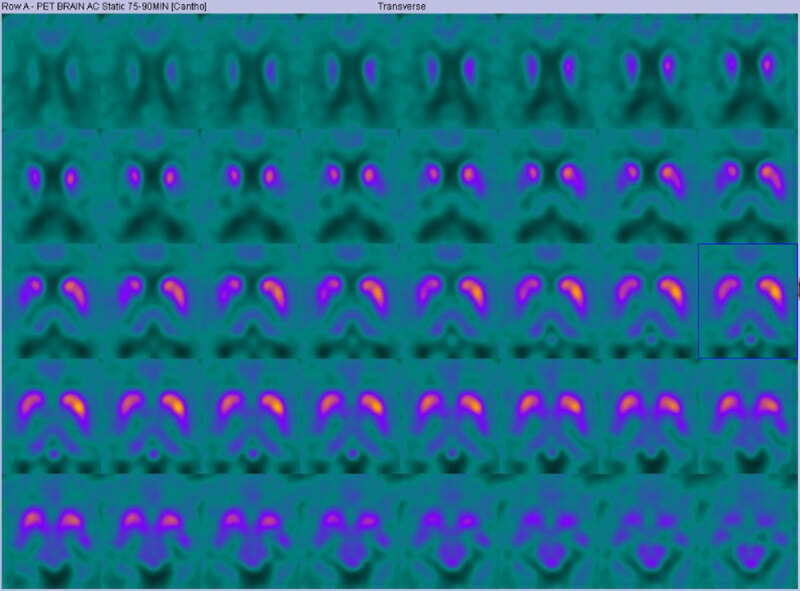
Image of ^18^F-DOPA PET/computed tomography (CT) of patient. Image demonstrates an asymmetric dopaminergic deficit (worse on the right side) suggestive of Parkinson’s Disease.

A 56-year-old woman, with no relevant medical or family history, was referred to our movement disorders service for a two-year-history of progressive tremors in her left hand. She was not using any medications and denied using illicit agents. On examination, she demonstrated a coarse left upper limb tremor at rest, as well as with posture and action. Tone was normal but mild bradykinesia was found on the left side, with reduced arm swing while walking. ^18^F-DOPA PET/computed tomography (CT) showed educed uptake in the right putamen (***Figure 1***) in keeping with a diagnosis of early-stage Parkinson’s disease and she was commenced on levodopa/benserazide.

**Figure 2:**
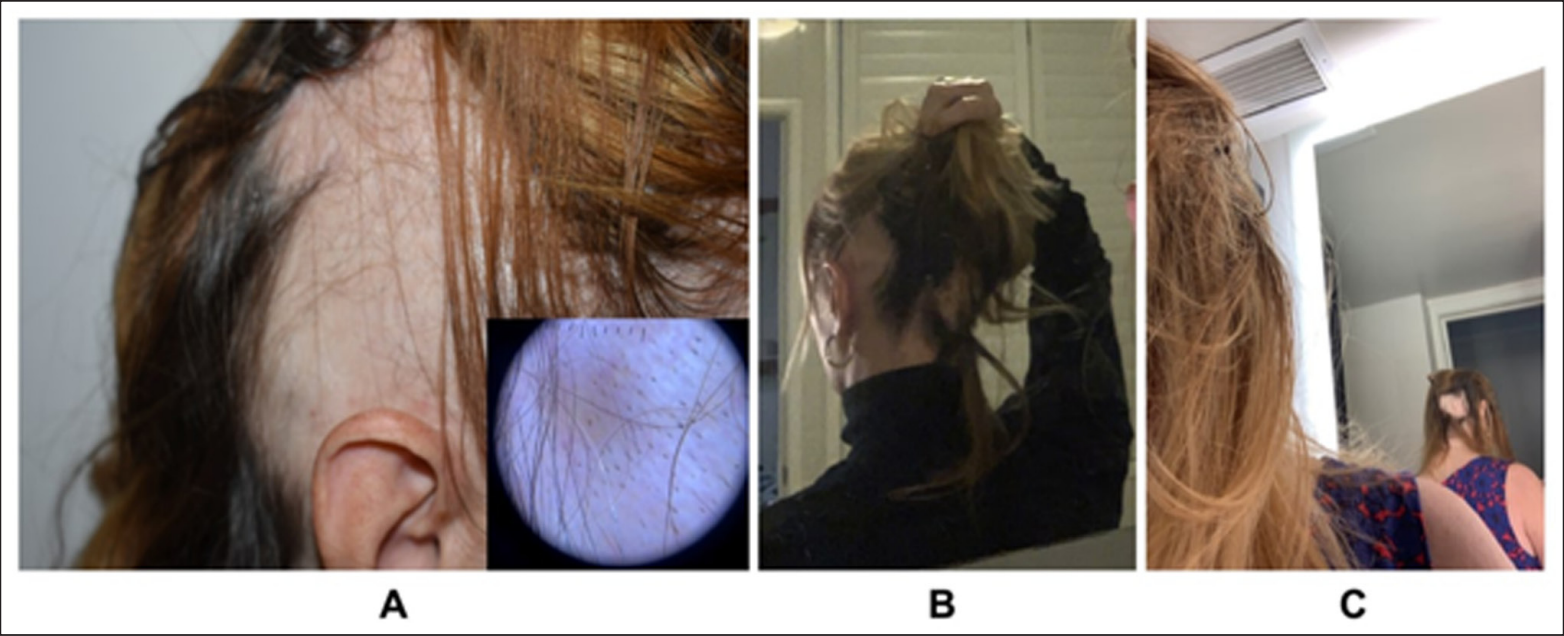
Drug-induced Alopecia Areata after **A)** Levodopa, **B)** Propranolol, and **C)** Topiramate.

Four weeks later, on a dose of 100/25 mg three times a day, she called in panic because of sudden severe hair loss on the right back of her head (see ***Figure 2a***). Dopaminergic medication was ceased immediately. Laboratory investigations were completely normal and on dermatology review she was diagnosed with alopecia areata, based on the pattern and speed of her patchy bald lesions. There were no signs of another underlying auto-immune disease. The patient was commenced on combination therapy topical steroids/calcineurin inhibitors/minoxidil and after three months she noticed slow regrowth of hair on the scalp.

Half a year later, our patient described worsening of her tremors when holding cups. She had also experienced reduction after alcohol consumption, and we trialed her on propranolol therapy. Initially, she reported an 50% improvement of tremulous movements, however, on a dose of 40 mg per day, she developed significant patchy hair loss again (see ***Figure 2b***), causing us to cease the beta-blocking agent. This time, regrowth occurred in a couple of weeks, and in the light of the good response on propranolol, we commenced her on a build-up scheme of primidone. Despite an excellent effect on her tremors, she had to be weaned off on a dose of 125 mg bi-daily because of cognitive dysfunction and early hallucinations.

One year later, her tremors had extended to her left leg, and after a long discussion we started her on a low dose of topiramate, 50 mg daily. Unfortunately, within less than two weeks the patient noticed first signs of alopecia on the back side of the scalp (see ***Figure 2c***) and after termination of topiramate, regrowth occurred once more after a couple of weeks.

## Discussion

Alopecia is a rare but debilitating side effect of various drugs used in tremor treatment. The term is derived from the Greek word for fox, “alopex,” and was so-named due to fur loss seen in fox mange. In humans, it most commonly affects the scalp resulting in patchy bald lesions, however any hair-bearing area might be involved. The hair loss may occur as a single, self-limiting episode or may reiterate at varying intervals over many years. Some people might even exhibit persistent bald areas and patches with regrowth simultaneously.

The normal hair growth cycle consists of three stages. The anagen phase refers to the period of 2–7 years of hair growth, in which follicles produce the entire hair shaft from tip to root. After a short transitional phase (catagen), hairs enter the final telogen or resting phase for a few months. At the end of this phase, the resting hairs fall out to allow new hair to start growing in the follicles. Hair loss in alopecia areata, has mostly been considered an immune-mediated disorder due to a lymphocytic attack on the hair bulb, although the exact pathophysiological mechanisms are not fully understood. It may occur in association with other auto-immune disorders (especially thyroid disorders) but is also commonly seen after chemotherapy and radiation [1]. Multiple drugs have been proposed as contributors for this immune response as well. The inflammation is specific for anagen hair, causing disruption of the growing phase (*anagen effluvium*) resulting in sudden hair loss that occurs within days or weeks. However, if the inhibitory influence is removed, regrowth could happen quickly.

A second mechanism, *telogen effluvium*, describes the shedding of hairs due to premature entry of anagen follicles into the resting ‘telogen’ phase and can be caused by stress, childbirth, along with many pharmaceuticals.

Medication-induced hair loss has sporadically been described in Parkinson patients on levodopa [2] and other forms of dopaminergic therapy [3,4]. Except for one patient with alopecia of the beard, all cases of dopaminergic-induced hair loss occurred in women [5]. The fact that dopamine-1 receptor transcripts are present in human hair follicles is suggestive for a mediating role and a more recent studies provided evidence for dopamine functioning as catagen promoter, pushing hair follicles from the anagen to the telogen phase, and causing regression of hair growth [6]. However, the exact pathophysiological relation between of dopamine and alopecia remains unclear.

Other drugs associated with alopecia include beta-blocking agents. Propranolol is one of several beta-blockers that can cause hair loss, however, given the widespread clinical usage, cases have been rare [7]. The suggested mechanism is a direct toxic effect on hair follicles [8]. The hair loss from propranolol is not permanent and is typically a result of telogen effluvium.

Cosmetic side effects, as is alopecia, are one of the most frequently reported side effect categories in anti-epileptic drugs (AED) and have a high intolerability rate. Hair loss has been described in valproate, carbamazepine, topiramate, phenytoin, phenobarbital, and clonazepam. Most cases have labelled telogen effluvium as the type of hair loss in AED, especially in patients taking valproate or carbamazepine [9]. Topiramate-induced alopecia is reported to occur in 1–2% of its users [10]. In most case reports, hair loss was reversible upon discontinuation or reduction of the dose. Recurrence of alopecia after reintroduction has also frequently been described.

Initially, our tremor patient developed alopecia areata after commencement of levodopa. After hair regrowth, both we and the patient, were reluctant to trial her on other forms of dopaminergic therapy and due to the significant action- and postural component of her tremors, we considered the possibility of a mixed tremor type (Parkinson’s tremor with essential tremor overlap). Therefore, our next step was to commence her on propranolol and later topiramate, but unfortunately alopecia reoccurred in both cases. The time duration between drug-introduction and onset of hair loss differed between four days to three months. For all three medications, hair loss was reversible after discontinuation and regrowth occurred in a few weeks. No other causes for alopecia were found. After cessation of topiramate, she decided to withhold from further medication options. As her tremors have worsened, she is currently under consideration for Deep Brain Stimulation surgery.

Alopecia areata is a significant adverse effect of different types of medication used in the treatment of tremors and can have a negative impact on emotional well-being and social functioning. Fortunately, in most cases, as well as in our patient, the hair loss is reversible after reduction or cessation of the causing agent.
